# An open-source pipeline for analysing changes in microglial morphology

**DOI:** 10.1098/rsob.210045

**Published:** 2021-08-11

**Authors:** Devin Clarke, Hans S. Crombag, Catherine N. Hall

**Affiliations:** School of Psychology and Sussex Neuroscience, The University of Sussex, Falmer, Brighton BN1 9QH, UK

**Keywords:** microglia, morphology, *in vivo*, two-photon, image processing, dimensionality reduction

## Abstract

Changes in microglial morphology are powerful indicators of the inflammatory state of the brain. Here, we provide an open-source microglia morphology analysis pipeline that first cleans and registers images of microglia, before extracting 62 parameters describing microglial morphology. It then compares control and ‘inflammation’ training data and uses dimensionality reduction to generate a single metric of morphological change (an ‘inflammation index’). This index can then be calculated for test data to assess inflammation, as we demonstrate by investigating the effect of short-term high-fat diet consumption in heterozygous Cx3CR1-GFP mice, finding no significant effects of diet. Our pipeline represents the first open-source microglia morphology pipeline combining semi-automated image processing and dimensionality reduction. It uses free software (ImageJ and R) and can be applied to a wide variety of experimental paradigms. We anticipate it will enable others to more easily take advantage of the powerful insights microglial morphology analysis provides.

## Graphical abstract

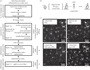

## 1. Introduction

Microglia, the brain's resident immune cells, are involved in phagocytosis and regulation of the adaptive immune response [1,2]. While resting they have a small soma and long, dynamic processes, allowing them to survey their local environment [3] for pathogens or damage-associated molecular patterns (DAMPs) [4]. Such stimuli activate a morphological shift towards an amoeboid-like shape which facilitates migration to sites of injury and phagocytosis [3,5]. To study such changes in morphology *in vivo*, transgenic animals expressing endogenous fluorescent reporters in microglia can be imaged through a cranial window over time (see [6] for a review of multiphoton *in vivo* imaging of microglia).

However, analysing morphological changes is often done manually. This can lead to rater error, demands a large time investment, and limits the number of cells that can be analysed. Furthermore, selecting which metrics of microglial anatomy to measure, from the number of branches to the overall shape of the soma, can be highly arbitrary. Although measuring many parameters simultaneously might better capture subtle variations, the multiple comparisons involved increase the chance of a false positive, while correcting for these decreases statistical power. With complex studies demanding large sample sizes to detect small effects, a more efficient approach is necessary.

As such, the three desirable features of a microglial morphology analysis pipeline are (i) automation to reduce rater error and time investment, (ii) measurement of multiple morphological features and (iii) reduction in the dimensionality of these features into a single index of morphological change.

Previously Kozlowski & Weimer [7] produced an automated pipeline for extracting morphological data from *in vivo* microglial images, but only measured a limited subset of features and did not make their methods publicly available. Work by York *et al*. [8] enabled researchers to take three-dimensional measurements of microglial cell stacks from both *in* and *ex vivo* datasets, but did not provide a solution regarding dimensionality reduction. Heindl *et al*. [9] went a step further by combining a range of measurements into a single metric that tracked microglial activation. However, their image segmentation work was not appropriate for use with *in vivo* images, in which motion artefacts need to be corrected, and they did not provide access to their dimensionality reduction process.

**Figure 1.**
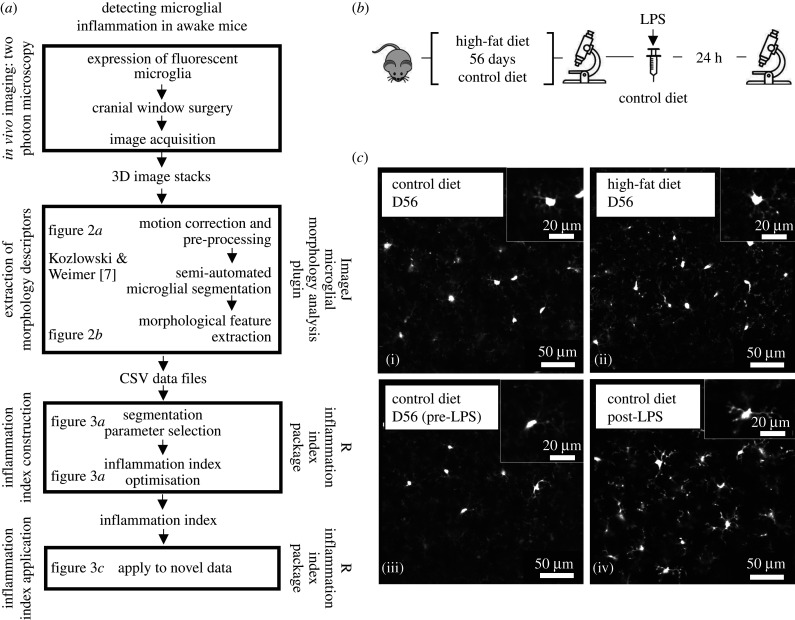
Experimental design. (*a*) Inflammation index construction and application schematic. (*b*) Imaging timeline. Mice were imaged after 56 days of control or HFD feeding. Following this, control mice were injected with LPS and imaged 24 h later. (*c*) Cx3CR1-GFP two-photon *in vivo* microglia images. Inset shows magnified view of example cells. (iii, iv): pre- and post-LPS microglia (training dataset). (i, ii): microglia after 56 days of control or HFD (test dataset). Main scale bar represents 50 µm. Inset represents 20 µm.

To address these limitations, we built an open-source microglial morphology analysis pipeline using both ImageJ and R (figure 1). We used our ImageJ plugin to process *in vivo* images of resting and bacterial lipopolysaccharide (LPS)-activated microglia from mice. The plugin cleans images, segments microglia from background and extracts 62 morphological features for each cell. Following this, we used our R package to combine the features best at discriminating between LPS conditions into a single ‘inflammation index’. Here, we demonstrate that this inflammation index provides a stable measurement of cellular morphology that persists over an imaging session, we use it to assess the impact of high-fat diet (HFD) feeding on microglial morphology in C-X3-C motif chemokine receptor 1-green fluorescent protein (CX3CR1-GFP^+/−^) mice, and we further validate our technique using *in vivo* images of two-pore domain K^+^ channel THIK-1 (TWIK-related halothane-inhibited K^+^ channel) knockout microglia [10]. Researchers can download the two packages necessary to perform this whole analysis pipeline from the GitHub repository (https://github.com/BrainEnergyLab/Inflammation-Index).

## 2. Material and methods

First, we describe the experimental protocols used to generate our *in vivo* microglia datasets, before describing the detailed analysis pipeline, employing the ImageJ plugin and R package, that allows the level of microglial inflammation to be measured based on changes in their morphology (figure 1).

### 2.1. Data acquisition

#### 2.1.1. Animals

Mice (*Mus musculus* Linnaeus) were housed individually in a temperature-controlled room (22 ± 2°C) with a 12 h light/dark cycle. A group of 10 mice, 12–18 weeks of age and of both sexes, were used with five mice fed a control chow diet (Research Diets D12450H-150FBZ, New Brunswick, NJ, USA) and the other five fed a HFD (nutrient balanced with 45% of total calories from fat, Research Diets D12451-150FBZ). Body weight was monitored daily. This sample size was chosen arbitrarily given we had no prior data based on inflammation index analyses to suppose an effect size. For diet group allocation, the first mouse that was available for imaging after cranial window surgery was allocated to the control group, and thereafter we alternated diet allocation for each next-available mouse. Data collected from these 10 mice were used in the test dataset, while data collected from the five control mice in pre- and post-LPS conditions formed the training dataset.

Mice were C57/BL6 and heterozygotes for Cx3CR1-GFP, so microglia expressed GFP [11]. Heterozygotic mice were used as Cx3CR1-GFP homozygous mice are known to show a detrimental phenotype being resistant to the beneficial effects of environmental enrichment on neuronal plasticity, CA1 and hippocampal long-term potentiation, and Morris Water Maze performance [12].

#### 2.1.2. Surgical window implantation procedure

Cranial windows were implanted to enable two-photon imaging of microglia in the primary visual cortex (V1). Mice were anaesthetized in an induction chamber with 4% isoflurane (IsoFlo, Zoetis UK Limited, Leatherhead, Surrey, UK) until their breathing rate reached 1.5 Hz at which time the isoflurane concentration was decreased to 2% and the induction chamber was flushed for 4 s. The animals were then secured in a stereotactic frame (Kopf Instruments, Tuunga, CA, USA). Reflexes were checked to ensure adequate anaesthesia levels before administering subcutaneous injections of 0.9% saline (400 µl), the opioid analgesic buprenorphine (1*.*2 µg, 0.3 mg ml^−1^, Vetergesic, Ceva Animal Health, Amersham, Buckinghamshire, UK), the anti-inflammatory steroid dexamethasone (120 µg, 2 mg ml^−1^, Dexadreson, MSD Animal Health, Milton Keynes, Buckinghamshire, UK) and the non-steroidal anti-inflammatory meloxicam (6*.*2 µg, 0.5 mg ml^−1^, Metacam, Boehringer Ingelheim Animal Health, Bracknell, Birkshire, UK) to reduce dehydration, post-operative pain and inflammation, respectively. The temperature was maintained at 37°C throughout using a homeothermic blanket (PhysioSuite, Kent Scientific Corporation, Torrington, CT, USA).

Hair over the skull was first trimmed using scissors, before the remaining hair was removed with hair removal cream (Veet, Reckitt Benckiser, Slough, Berkshire, UK). The exposed skin was cleaned with saline, then ethanol and finally iodopovidone (Betadine, Mundipharma, Cambridge, Cambridgeshire, UK). The skin and periosteum over the skull were cut away and removed with small spring scissors and forceps (Fine Science Tools, Heidelberg, Germany) across the entire dorsal skull surface. During this process, any bleeding was stemmed using absorption spears (Fine Science Tools). The edges of the skin were then sealed to the skull with surgical cyanoacrylate (Vetbond, 3M, Bracknell, Berkshire, UK) before surgical calipers and a pen were used to mark the location of the craniotomy, as a circle with a 3 mm diameter overlaying the visual cortex (centred on a point 3.10 mm lateral to lambda and 1.64 mm anterior of the lambdoid suture). The skull, excluding the marked region, was then roughened using a scalpel to create overlapping scores in perpendicular directions to aid cement and head plate adhesion, before being covered in surgical cyanoacrylate. The mouse was then tilted on the head mount so that the area marked for the craniotomy lay flat. The roughened area was covered with dental cement (Unifast TRAD, GC Europe, Leuven, Belgium; previously mixed with black acrylic), and a custom-built stainless-steel head plate (University of Sussex workshop, Brighton, East Sussex, United Kingdom) was placed over the dental cement and left for a few minutes until dry.

Following this, a dental drill (OmniDrill 35, World Precision Instruments, Sarasota, FL, USA) was used to drill around the edges of the marked area alternating between 0.5, 0.7 and 1 mm drill bits as necessary (diameter at the tip; Fine Science Tools) ensuring that regular breaks were taken and that the area was frequently irrigated with saline to prevent overheating of the brain (temperature increases at the brain's surface of 25°C can result from continuous drilling and are associated with significantly increased blood–brain barrier permeability. This did not occur when regular breaks were taken; [13]. The area surrounding the marked area was also flattened. Once the skull was thin enough, the bone was moistened with saline for a final time and then lifted off with forceps and a microprobe. Gelfoam (Pfizer, New York, NY, USA) was used to stop any bleeding of the dura. Once the bleeding was halted, forceps were used to remove the dura (if still present after the craniotomy).

An optical window (made from two 3 mm glass coverslips and a 5 mm glass coverslip (Harvard Apparatus, Holliston, MA, USA) sealed with optical adhesive (Norland, Cranbury, NJ, USA)) was placed into the craniotomy and secured using a glass rod while absorption spears were used to dry the liquid surrounding the window. The edges of the glass window were then sealed to the skull, first with surgical cyanoacrylate, then with dental cement. Finally, two rubber rings were secured on top of the head plate with dental cement to serve as a water reservoir during two-photon imaging using a water-based objective. Anaesthesia was removed and mice were placed into an incubator (37°C) to waken, before being singly housed in a recovery cage. For 3 days following the surgery mice were given daily 10 µg doses of meloxicam mixed into wet food mash.

#### 2.1.3. *In vivo* experimental set-up

Beginning at least two weeks after surgery mice were gradually habituated to the imaging rig. Habituation reduces both excessive movement during image collection and chronic restraint stress, where the latter can influence microglial morphology [14]. *In vivo* images were collected 12 weeks after surgery, well after astrocytic and microglial responses to the surgery had subsided (30 days after V1 cranial window surgery for astrocytes, and after 7–10 days for microglia [15]). During imaging sessions, mice were head-fixed underneath a two-photon microscope (Scientifica, Uckfield, UK) atop of a free-moving polystyrene cylinder (Biosciences Workshop, UCL) fitted with a rotary encoder (Kubler Group, Villingen-Schwenningen, Germany) that allowed recording of voluntary running. Imaging sessions were performed in the dark. Immediately prior to imaging sessions, the vascular lumen was labelled either by intravenous injection with 2.5% (w/v) Texas Red Dextran (70 kDa, Invitrogen, Carlsbad, CA, USA) or subcutaneous injection of 2.5% (w/v) Texas Red Dextran (3 kDa, neutral, Invitrogen).

#### 2.1.4. Two-photon microscopy

High-resolution imaging of microglia was performed with a commercial two-photon microscope (Scientifica, Uckfield, UK) using a high numerical aperture water-immersion objective (Olympus 20X XLUMPLFLN20XW, Shinjuku City, Tokyo, Japan) with a working distance of 2 mm and a mode-locked Ti-sapphire infrared laser (Chameleon, Coherent, Santa Clara, CA, USA). The tissue was excited at a 940 nm wavelength, and the emitted light was filtered to collect green light from GFP (Cx3CR1-GFP-labelled microglia). Imaging sessions were recorded using SciScan software (Scientifica). Laser power at the objective was kept below 25 mW to minimize photodamage. Laser power and photodetector gain was kept relatively constant between imaging sessions to minimize variations in the signal-to-noise ratio of images.

To capture data for the purpose of analysing microglial morphology, stacks sampling 297 × 297 × 100 µm of tissue (*x*, *y* and *z* dimensions, respectively) were collected from 50 to 150 µm below the window at a resolution of 512 × 512 pixels with a voxel size of 580 × 580 × 1000 nm (*x*, *y*, *z*). Six frames were taken at each imaging plane, and image stacks were saved as single-channel stacks in .tif format. Imaging parameter choices were based on Kozlowski & Weimer [7].

In addition, we also acquired four-dimensional image stacks for the purpose of tracking microglial morphological changes within an imaging session. These images sampled 138 × 138 × 52 µm of tissue (*x*, *y*, *z*) and were collected from 50 to 100 µm below the window at a resolution of 512 × 512 pixels with a voxel size of 270 × 270 × 2000 nm (*x*, *y*, *z*). We captured 14 frames per plane and acquired two stacks for each treatment condition. The stacks were acquired 18 min apart.

#### 2.1.5. Lipopolysaccharide-Induced microglial activation

Around 12 weeks after cranial window surgery, mice were given intraperitoneal injections of LPS (O111:B4; Sigma-Aldrich, St Louis, MO, USA) at a dosage of 4 mg kg^−1^ in 100 µl of 0.9% saline to induce microglial activation and imaged 24 h later. Following the imaging session, mice were culled using a terminal dose of intraperitoneal sodium pentobarbital (Dolethal; Vétoquinol UK Ltd, Towcester, Northamptonshire, UK) at 120 mg kg^−1^ (diluted to 10% in saline).

#### 2.1.6. Dietary manipulation

Dietary manipulation began no sooner than four weeks after cranial window surgery. Mice were fed either a control diet or HFD for 56 days. Microglial images from V1 were acquired the day before treatment began and then again on day 56 of dietary manipulation. Control diet mice were subsequently injected with LPS as described above.

### 2.2. Image processing and analysis

Images were processed and analysed using the Fiji distribution of ImageJ [16]. By having a third party rename animal identifiers in the file and folder names, all image processing was done blind to mouse identity and dietary or LPS treatment. The custom-written ImageJ plugin (Microglial Morphology Analysis; https://github.com/BrainEnergyLab/Inflammation-Index) performs all image processing steps as described below, which are automated except where indicated. Details of how to use the plugin, including the file structure required, can be found in the README file (https://github.com/BrainEnergyLab/Inflammation-Index/blob/master/Using%20the%20Microglia%20Morphology%20Analysis%20ImageJ%20Plugin.md).

#### 2.2.1. Image preprocessing

**Figure 2.**
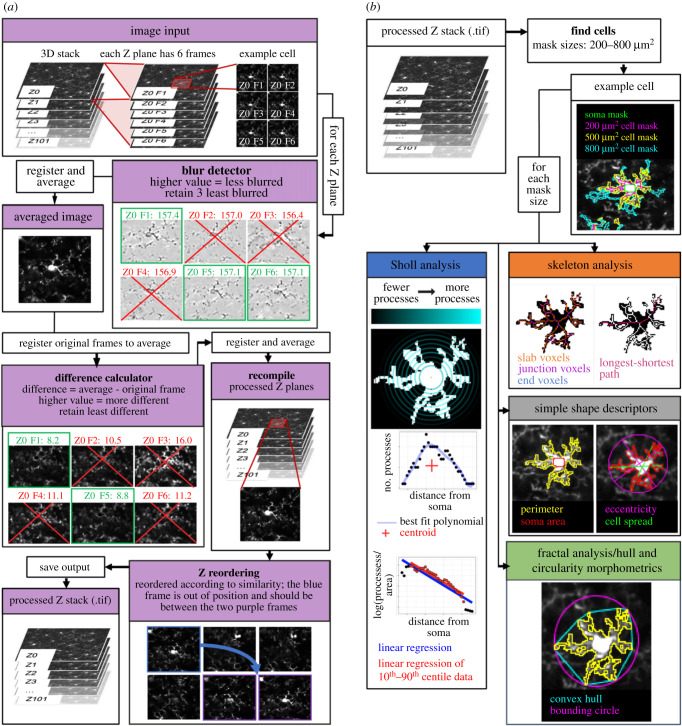
Image processing and extracting morphological descriptors. (*a*) Image processing schematic. Input stacks were split into substacks containing all frames from a distinct Z level. For each substack, the least blurry frames were registered and averaged to create a reference frame that each frame of the substack was then registered to. The frames least different from the reference were again registered and averaged into a single frame. These single frames were stacked and reordered to represent their true positioning in Z. (*b*) Morphological feature extraction. After generating cell masks for a user-specified range of target mask sizes, the ImageJ plugin measured 62 different morphological features for each cell. These features were derived from five domains: simple shape descriptors, skeleton analyses, fractal analyses, hull and circularity morphometrics, and Sholl analyses.

First, two-photon images needed to be preprocessed to improve image quality and remove motion artefacts as mice were imaged when awake (figure 2*a*). This pipeline describes the processing of signal-channel images. Performance maybe enhanced if parallel images of vasculature are used to register the vascular channel with the ‘Register Virtual Stack Slices' ImageJ plugin, and the output containing the applied transformations is then applied to the microglial channel using the complementary ‘Transform Virtual Stack Slices’ plugin.

In our pipeline, the order of the frames in the input stacks were of the structure F1Z1, F2Z1, F3Z1, F1Z2, etc., where each F denotes a single frame captured at each Z depth.

(a) To enhance the image clarity, contrast levels within a three-dimensional stack were normalized using the stack contrast adjustment plugin [17] before the stack was divided into substacks of frames captured at each Z level (e.g. our three-dimensional image stacks represented 100 µm of tissue and were split into 100 substacks, each containing six images).
(b) Frames distorted by motion during image acquisition were then removed from each Z plane substack. Each frame within a substack was passed through a blur detector, where it was subject to a Laplacian of Gaussian (LoG) filter from the FeatureJ suite of ImageJ plugins [18], and the standard deviation of the pixel intensity in the resulting image was measured. This filter highlights regions of an image containing rapid intensity changes (typically edges), with the standard deviation of the intensity in the resulting image an indicator of sharpness (where blurred images tend to have fewer edges). Using this method, the three least blurry frames in each substack were identified (users can set this number as they wish to fine tune their preprocessing steps). These were then registered to each other using the translation method in the MultiStackReg ImageJ plugin [19] and averaged by creating a mean intensity projection of the three frames to yield a minimally blurry single image for each Z plane that served as a reference frame for detecting motion between frames within the Z plane substack. Frames with significant jitter were detected by registering each frame within the original substack to the reference frame (using the translation method in MultiStackReg) and calculating the mean difference between pixels in the reference image and each frame of the newly registered original substack using the Image Calculator functionality in ImageJ. The two least different frames were retained for each Z substack and were registered to each other (using the translation method in MultiStackReg; users can set this number as they wish). They were then averaged by creating a mean intensity projection to generate a cleaned frame for each Z plane. These cleaned images were recompiled to create a preprocessed and motion cleaned version of the input three-dimensional stack, now composed of single frames representing each Z plane. The general approach applied in this step (i.e. selecting the least different frames relative to a reference frame for different Z substacks) was inspired by the work of Soulet *et al*. [20].
(c) Because movement in Z during image acquisition can mean that the actual Z location of acquired frames can differ from their apparent location, we next reordered each Z frame in the recompiled stack according to their actual Z location using the Z-spacing correction plugin [21].
(d) Stacks were then inspected manually to detect failures in preprocessing (e.g. where Z reordering in step (c) had failed, or where significant movement in the three-dimensional stack was still present). In these cases, the least blurry and motion-distorted frames at each Z plane were selected manually from the original input three-dimensional stack before being recompiled and registered. Where this manual selection was still not adequate (e.g. in cases where there was such severe movement during image acquisition that combining the highest quality frames for each Z plane still yielded too much movement or blurriness in the final stack), images were excluded from further analysis. These manual ‘quality control’ and frame selection steps are streamlined in the ImageJ plugin to make them as easy as possible for users.

#### 2.2.2. Semi-automated microglial segmentation and quantification

The methodology used to semi-automatically generate microglial cell masks from two-photon image stacks is based on the work of Kozlowski & Weimer [7]. As with the image preprocessing steps, all the following steps were run using the custom-written ImageJ plugin: microglial morphology analysis. In essence, these steps build on the methodology described by Kozlowski & Weimer [7] but extract a much greater array of morphology measurements from segmented cells. This process is built into the automated pipeline, with any manual input needed indicated clearly in the following steps.

(a) Identification of cells: our z-stacks were split into 10 µm substacks separated by 20 µm (preventing the same cells being sampled in multiple substacks). Frames within each substack were averaged using a mean intensity projection. Cell locations were detected automatically (by identifying maxima in these projections using the Find Maxima plugin in ImageJ) before being either manually approved, or manually edited and approved, on these projections. Following this, a 120 × 120 µm region of interest (ROI) was drawn around each marked cell. The ROIs were thresholded with the ImageJ thresholding plugin using Otsu's method [22] to find the initial thresholding value. All pixels above this value that were in contact with the marked cell locations within each ROI were then used to form a cell mask by implementing the Find Connected Regions plugin in ImageJ [23]. The area of this mask was calculated and then compared to a user-defined target area (from here on referred to as mask size). If the measured area of the mask matched the user-defined mask size (within user-defined limits—we used ±100 µm^2^), the mask was considered complete. Otherwise, iterative thresholding was used whereby the applied threshold value was adjusted until either the measured mask area fell within the desired range, or the mask area stabilized for three consecutive iterations. If after passing these criteria, a mask was within 5 µm of the edge of the ROI, it was rejected. The formula used to determine the threshold for the next iteration relates the threshold for the next iteration (*T_I_*_+1_) to the current threshold (*T_I_)*, the area of the current threshold (*A_I_*), the mask size (*MS*) and the number of iterations thus far (*n*):
  2.1 $$T_{I+1}=T_{I}+\;T_{I}\left(\frac{A_{I}-MS}{n(MS)}\right).$$
  Cell masks were excluded from further analysis if an accepted mask contacted the edge of the 120 × 120 µm ROI.
(b) Approval of cell masks: after automated mask generation was completed, each mask was overlaid on its original image and visually inspected to determine if it included processes from adjacent cells. If so, these masks were excluded from further processing in the pipeline. This step is semi-automated in the ImageJ plugin to reduce the time required.
(c) Soma detection: for accepted masks, the cell soma were identified by first thresholding the ROIs using Otsu's method [22], then excluding particles that were smaller than 20 µm^2^ with a circularity value of less than 0.6. If a single particle remained, this was identified as the cell soma. These automatically generated soma masks were then subjected to user assessment, where if the mask was rejected, or automatic detection failed to identify a soma mask, a manual soma mask was drawn.
(d) Measurement of morphological characteristics: multiple parameters were calculated to capture different aspects of microglial morphology using a variety of Image J plugins (figure 2*b*). The Sholl Analysis plugin was used to perform Sholl analyses [24]; the built-in ImageJ measurement functions were used to extract simple shape descriptors (for example perimeter length, circularity); the built-in Skeletonize plugin and the AnalyzeSkeleton plugin [25] were used to analyse the skeletonized cell mask by, for example, calculating branch lengths, area occupied by the skeletonized cell and the number of junction voxels in the skeleton, and FracLac [26] was used to extract fractal, and hull and circularity morphometrics. The full list of extracted features is given in table 1.

**Table 1.** The 62 morphological features extracted by the ImageJ plugin.

descriptor	type	definition
perimeter	simple	perimeter around the cell mask
cell spread	simple	average distance from the centre of mass of the mask to the four extremities
eccentricity	simple	ratio of the major and minor axes of an ellipse drawn around the mask
roundness	simple	inverse of the eccentricity
soma area	simple	area occupied by the microglial soma mask
mask area	simple	area of the cell mask
branches	skeleton	the number of branches in the mask skeleton
junctions	skeleton	the number of junctions in the mask skeleton
endpoint voxels	skeleton	the number of endpoints voxels in the mask skeleton
junction voxels	skeleton	the number of junction voxels in the mask skeleton
slab voxels	skeleton	the number of slab voxels in the mask skeleton
average branch length	skeleton	the average length of all branches in the mask skeleton
triple points	skeleton	the number of junctions with 3 branches
quadruple points	skeleton	the number of junctions with 4 branches
maximum branch length	skeleton	the maximum length of a branch in the skeleton
longest shortest path	skeleton	the sum of the shortest path between all pairs of vertices
skeleton area	skeleton	area occupied by the cell skeleton
primary branches	Sholl	number of branches originating directly from the cell soma
intersecting radii	Sholl	the number of sampled radii with at least one intersecting process
sum of intersections	Sholl	the total number of processes
mean of intersections	Sholl	the mean number of processes across all sampled radii
median of intersections	Sholl	the median number of processes across all sampled radii
skewness (sampled)	Sholl	the skewness of the distribution of the number of branches
skewness (fit)	Sholl	the skewness of the distribution of the number of branches derived from the best fit polynomial
kurtosis (sampled)	Sholl	the kurtosis of the distribution of the number of branches
kurtosis (fit)	Sholl	the kurtosis of the distribution of the number of branches derived from the best fit polynomial
maximum number of intersections	Sholl	the highest number of processes at any given radius
max intersection radius	Sholl	the radius at which the max intersections occurs
ramification index (sampled)	Sholl	the ratio between the max intersections and the number of primary branches
ramification index (fit)	Sholl	the ratio between the max intersections and the number of primary branches based on the best fit polynomial
centroid radius	Sholl	the abscissa of the geometric centre of a linear plot of number of branches against radius
centroid value	Sholl	the ordinate of the geometric centre of a linear plot of number of branches against radius
enclosing radius	Sholl	the largest sampled radius
critical radius	Sholl	the local maximum of the polynomial fit
mean value	Sholl	the mean value of the polynomial fit
polynomial degree	Sholl	the degree of the best fit polynomial
regression coefficient (semi-log)	Sholl	the slope of the linear regression between the log of branch number plotted against sampled radius
regression coefficient (semi-log)[P10-P90]	Sholl	the slope of the linear regression between the log of branch number plotted against sampled radius but only for data between the 10th and 90th percentiles
regression coefficient (log–log)	Sholl	the slope of the linear regression between the log of branch number plotted against the log of the radius
regression coefficient (log–log)[P10-P90]	Sholl	the slope of the linear regression between the log of branch number plotted against the log of the radius but only for data between the 10th and 90th percentiles
regression intercept (semi-log)	Sholl	the intercept of the linear regression between the log of branch number plotted against sampled radius
regression intercept (semi-log)[P10-P90]	Sholl	the intercept of the linear regression between the log of branch number plotted against sampled radius but only for data between the 10th and 90th percentiles
regression intercept (log–log)	Sholl	the intercept of the linear regression between the log of branch number plotted against the log of the radius
regression intercept (log–log)[P10-P90]	Sholl	the intercept of the linear regression between the log of branch number plotted against the log of the radius but only for data between the 10th and 90th percentiles
density	HC morph.	mask area divided by the area of the convex hull
span ratio	HC morph.	ratio of the major to minor axes of the convex hull
maximum span across hull	HC morph.	the maximum distance across the convex hull
convex hull area	HC morph.	area of the convex hull
convex hull perimeter	HC morph.	perimeter of the convex hull
convex hull circularity	HC morph.	circularity of the convex hull
maximum radius from hull's centre of mass	HC morph.	mean length from the centre of the convex hull's mass to points on the convex hull
max/min radii	HC morph.	the ratio of the largest to smallest radius from the centre of mass of the convex hull to an exterior point
CV for all radii	HC morph.	the coefficient of variation in the length of the radii from the centre of mass of the circle to points on the convex hull
mean radius	HC morph.	the mean length from the centre of mass to an exterior point on the convex hull
diameter of bounding circle	HC morph.	the diameter of the smallest circle enclosing the convex hull
maximum radius from circle's centre	HC morph.	mean length from the centre of the minimum bounding circle to points on the convex hull
max/min radii from circle's centre	HC morph.	the ratio of the largest to smallest radius from the centre of the minimum bounding circle to an exterior point
CV for all radii from circle's centre	HC morph.	the coefficient of variation in the length of the radii from the centre of the minimum bounding circle to points on the convex hull
mean radius from circle's centre	HC morph.	the mean length from the centre of the minimum bounding circle to an exterior point on the convex hull
fractal dimension	fractal	a measure of complexity of the cell shape, i.e. how a pattern's detail changes with the scale at which it is considered
lacunarity	fractal	a quantification of the inhomogeneity in the cell mask, also understood as a measure of ‘gappiness’, visual texture, and translation and rotational invariance
branching density	—	skeleton area divided by the convex hull area

### 2.3. Composite morphology measure construction

To best compare inflammation in different conditions, we combined all these measures into a single inflammation index, using an approach based on work by Heindl *et al*. [9]. This method identifies the morphological measures which best discriminate between conditions and passes these measures through a principal component analysis (PCA) to generate a single index of morphological change. In our pipeline, this process takes place using training data (in our case, pre- and post-LPS measurements), and the weights applied to the best discriminators to generate the single index are then applied to the test data (in our case, control and HFD measurements) to evaluate if test conditions lead to significant changes in this morphological index (in our case, an index that reflects how similar to LPS-activated microglia a cell's morphology is). However, as the data extracted from our images depend on the user-defined mask size, the results of this process will depend on what size is selected. Because of this, we first ran our image analysis steps on our pre- and post-LPS images for a range of mask sizes before constructing the composite index for each mask size. We then selected the optimal mask size for discriminating pre- and post-LPS cells and based our inflammation index on the index constructed using this mask size. We then extracted data from our test dataset (control and HFD images) using this mask size and applied the inflammation index to this. For this reason, inputting a greater range of mask sizes on which to run the initial microglial segmentation step maximizes the likelihood of creating a final inflammation index that is optimally sensitive to the training data conditions (in our case, pre- and post-LPS). Our R package ‘Inflammation-Index’ provides simple functions that users can employ to replicate this process, and (like our ImageJ plugin) is available at https://github.com/BrainEnergyLab/Inflammation-Index. The README for this package, including example inputs and outputs, can be found at https://github.com/BrainEnergyLab/Inflammation-Index/blob/master/Using%20the%20R%20Inflammation-Index%20Package.md. The R package uses the .csv results files output by the ImageJ plugin. As in the ImageJ plugin, most steps are automated, with some input of user-specified arguments required. Only images from pre- and post-LPS-treated mice, previously fed a control diet, were analysed for the construction of our inflammation index.

#### 2.3.1. Mask size selection (figure 3*a*)

**Figure 3.**
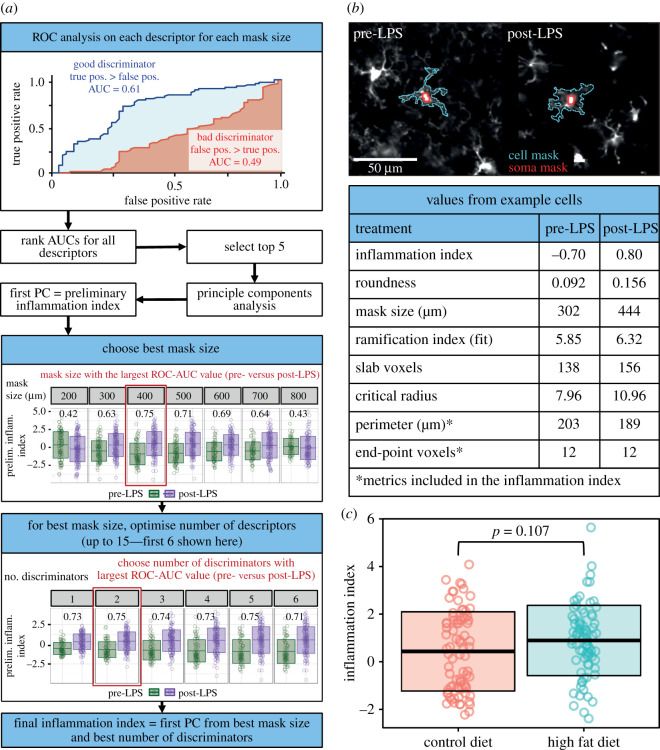
Calculating and applying the inflammation index. (*a*) For each mask size, the morphological descriptors' ability to discriminate between pre- and post-LPS conditions was evaluated using a ROC-AUC analysis (red, regression coefficient; blue, critical radius). A PCA was run on the top five discriminators. The first principal component compared pre- and post-LPS again using a ROC-AUC analysis. The mask size with the largest AUC value was selected. PCAs were then run on a range of the best discriminators at this mask size. The range (e.g. the best four discriminators) with the largest AUC value for the ability to discriminate between pre- and post-LPS was selected as the inflammation index. (*b*) Two example cells from the LPS dataset displaying values from the seven best discriminators. (*c*) This inflammation index can then be calculated for experimental data. It was unaffected by 56 days of HFD feeding (control mean ± s.d. of 0.44 ± 1.66; HFD mean ± s.d. of 0.90 ± 1.47). Dots are cells, lines represent the mean ± 1 s.d. Seventy-nine cells from 5 control mice, 77 cells from 5 HFD mice. Statistical tests were conducted using linear mixed models with the animal ID specified as a random intercept to avoid pseudoreplication.

(a) We ran our ImageJ plugin on our training data for a range of mask sizes (we used 200, 300, 400, 500, 600, 700 and 800 µm^2^ within limits of ±100 µm^2^). For each mask size, receiver operating characteristic (ROC) analyses were conducted for each morphological measure to determine their ability to discriminate between activated and resting microglia (pre- and post-LPS treatment). ROC curves plot the false-positive rate against the true-positive rate of binary classification (i.e. pre- and post-LPS) at differing thresholds of a metric (i.e. for a morphological measure such as cell perimeter, we plot the false-positive and true-positive rates of classifying a cell as pre- or post-LPS at a given threshold of cell perimeter. We do this for all possible thresholds for our cell perimeter measurements). The area under the curve (AUC) of these plots tells us how well different measures discriminate between pre- and post-LPS microglia.
(b) A selection of the most accurate discriminators based on their ROC-AUC values were retained. In this analysis, we limited our selection to the best five to ensure we only included measures that were good discriminators, though users can set the number of measures to retain themselves and this number is optimized in later steps. The choice to use a set number of measures, rather than a set value of the AUC as a cutoff, is discussed later. A PCA with feature centring and scaling was run on these measures, with the first principal component (which by definition is the weighting of the inputs that best captures the variability in the data—in this case, variability due to inflammatory state) serving as an initial inflammation index for each mask size.
(c) Several of our measures capture very similar features of a cell's morphology. For example, some Sholl parameters are calculated multiple times for different subsets of the data (e.g. 10th–90th percentiles versus the whole dataset), while hull and circle morphometrics are calculated based on both the centre of the bounding circle, and the centre of mass. In these cases, if multiple variants of the same metric were ranked as high discriminators, only the best performing variant was selected for inclusion in the PCA.
(d) Similarly, some measures are highly correlated with one another. Following the removal of low-performing metric variants, we removed highly correlated metrics. For a given pair of highly correlated measures, the measure with the lowest performance as a discriminator (according to the ROC-AUC analysis) was dropped. This process was repeated until the remaining selected metrics did not show correlations with one another above our threshold (a Pearson's correlation coefficient ≥ 0.9).
(e) We then selected the mask size which most significantly discriminated between pre- and post-LPS conditions. The ‘Inflammation-Index’ R package allows two ways to identify the best mask size. The first, which we used for the analyses presented here, compares the AUC values from a ROC analysis run on the inflammation index that evaluates its ability to discriminate between training conditions (pre- and post-LPS) for each mask size (figure 3*a*). The second option instead compares *p*-values for the effect of training conditions on the inflammation index. This comparison is done using a linear mixed model, with the animal identifier specified as a random intercept. For our data, the strongest effect of LPS was observed using a mask size of 400 µm^2^ . This optimal mask size is detected automatically within the ‘Inflammation Index’ R package, so all the following steps are performed only on data extracted using this mask size.

#### 2.3.2. Inflammation index optimization and application

Following mask size selection, the next steps in the pipeline first refine the inflammation index to be optimally sensitive to the morphological changes associated with training conditions (while avoiding it being overly specific to the data it is based on). The optimized inflammation index can then be applied to novel data to test for any activation-associated microglial morphological change. Again, these steps are all automated though can be adjusted by user input where indicated.

(a) Refining the inflammation index: the preliminary inflammation index is composed of a subjective number of the most accurate discriminating morphological measures (we used five). To optimize the inflammation index, we determined whether combining more, or fewer, measures produced a stronger effect of the training conditions (and therefore better discriminability between them). For the chosen mask size, the index was reconstructed using the best 1 to *N* discriminators of activated microglia using ROC curves as described above, where *N* is the user-defined maximum number of discriminators to be included. We used a maximum of 15 factors to avoid building an overly specific metric to the pre- and post-LPS data it was based on, and which therefore would be unfit for use with novel datasets. Inflammation indices derived using these different combinations of factors were then generated using a PCA as above and the best index was then identified as above by comparing the AUC values from ROC analysis run on the LPS treatment conditions between indices (e.g. one constructed with the best three discriminators versus one constructed with the best four). The R package also offers users the ability to select this based on the *p*-values of the effect of training conditions for each index.
(b) This final inflammation index represents the first principal component of a PCA run on the optimal number of morphological discriminators. As such, its value is based on a weighted combination of the different discriminators that were included in its construction (for a comparison of two cells, illustrating the values of seven of the metrics identified as the best discriminators between pre- and post-LPS conditions, and the inflammation index, figure 3*b*). As long as the same morphological measures are present, the same weighted combination can be computed for any novel dataset acquired in a similar way, and an inflammation index can be calculated. This inflammation index then allows users to evaluate the activation level of their microglia in different experimental conditions. The construction of this index for novel data is done automatically through the ‘Inflammation Index’ R package. The nature of the training data will affect what sort of changes will be best detected. As described here, our inflammation index is optimized for detecting LPS-like microglial activation but the use of other training data could allow optimal detection of, for example, morphological differences between peri-infarct or contralateral microglia in stroke models (as in [9]), or, as we demonstrate in the results and discussion section, differences between THIK-1 knockout and wild-type microglial cells.

## 3. Results and discussion

We developed an open-source image analysis pipeline that facilitates the largely automated processing of large imaging datasets and the creation of a single metric of morphological change using dimensionality reduction to allow users to leverage *in vivo* microglial morphological analyses. This makes analysis easier, diminishes the effects of rater error on results, removes the need for assumptions about which metrics will be most sensitive to experimental manipulation, maximizes statistical power and minimizes false discovery rates. Our ImageJ and R packages are freely available at https://github.com/BrainEnergyLab/Inflammation-Index. A detailed description of their use is provided in the Material and methods section. Here, we describe the construction of an inflammation index with this pipeline using data collected from mice before and 24 h after LPS treatment (known to increase microglial activation). We then used this index to evaluate the inflammatory effects of 56 days of HFD feeding on microglial morphology [27–29]. In addition, we demonstrate that the value of the inflammation index remains stable for a given cell over the course of an imaging session, and we show the flexibility of our pipeline by applying it to images of THIK-1 knockout microglial cells as previously published in Madry *et al.* [10].

### 3.1. Image acquisition

Image stacks of GFP-labelled microglia were collected from V1 in awake CX3CR1-GFP^+/−^ mice using *in vivo* two-photon imaging (figure 1) the day before dietary manipulation began and after 56 days of either control diet or HFD consumption. After imaging on day 56, control mice were injected with LPS and imaged 24 h later. The quality of these stacks depended on image resolution, frame capture rate and motion during acquisition. For our primary morphology analysis dataset, we found a capture rate of around one frame per second with a resolution of 1.8 pixels per micron allowed us to acquire high-quality images despite the mouse's movement. Users of our plugins should trial different combinations of imaging parameters to produce the highest quality input given differences in imaging set-ups.

### 3.2. Image analysis

There are three broad stages to the analysis pipeline, which are described in full in the Material and methods section:

(1) cleaning of image stacks and extraction of morphology descriptors (figure 2);
(2) construction of a composite morphology index based on training data (our pre- and post-LPS images; figure 3*a* and *b*); and
(3) use of the composite index to assess morphological changes in test data (control and HFD images; figure 3*c*).

### 3.3. Image cleaning and extraction of morphology descriptors

We used our ImageJ plugin to preprocess three-dimensional image stacks and semi-automatically identify microglia and measure 62 morphological descriptors (figure 2).

#### 3.3.1. Correction of noise and motion contamination

For each Z location in a three-dimensional image stack, our plugin uses an average image of the least blurry frames to identify the least motion contaminated frames, before averaging the latter to generate a single cleaned image. These single images are recompiled into a Z stack that is reordered to correct for shifts in Z position during image acquisition. Where noise or motion artefacts were too substantial to be corrected with these methods, image stacks were excluded from further analysis to only include the most accurate microglial renderings.

**Figure 4.**
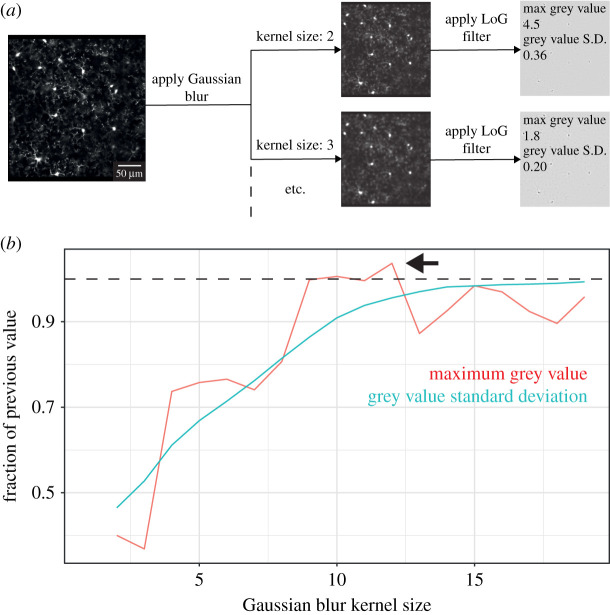
Comparison of methods for measuring blur in image frames. (*a*) Schematic of method comparison approach. A single image frame was blurred using different Gaussian kernel sizes (where larger kernels introduce more blur) before the blurred images were run through an LoG filter and their maximum grey value, and standard deviation of their grey values, were measured. Scale bar indicates 50 µm. (*b*) Plot of the maximum and standard deviation of different kernel sizes in the example image in (*a*). For each kernel size, values were divided by their value in the previous kernel size, and this fraction plotted. For every comparison with previous kernel size values, the standard deviation of the grey value was reduced. Conversely, the maximum grey value showed both increases (e.g. a greater value at kernel size 12 compared to 11; indicated by the arrow and the dotted line above which values are greater than at the previous kernel size) and decreases (e.g. a lesser value at kernel size 5 compared to 4). An ideal blur detector should show a consistent relationship with blur, and so the grey value standard deviation is a better tool for this than the maximum grey value.

Our approach is based on the work of Soulet *et al*. [20], who used an average projection of the frames in a Z plane as a template to detect, and remove, the frames in that plane that were most different from the template (i.e. contaminated by motion). We improved on this method by first adjusting the contrast across the entire stack, and then by using a blur detector to create our template using the least blurry frames. We implemented blur detection using the variance of the grey values of images subjected to a LoG filter based on previous methods [30], though we also analysed the effectiveness of using the maximum grey value to validate this approach (figure 4).

#### 3.3.2. Semi-Automated microglia segmentation and morphological feature extraction

Following image cleaning, we used our plugin to semi-automatically detect the location of microglia before generating cell masks using an iterative thresholding approach. Kozlowski & Weimer [7] used iterative thresholding to segment microglia and demonstrated that masks generated using iterative thresholding had approximately 90% similarity in terms of the numbers of processes counted when compared to manual results. Given that iterative thresholding requires a user-defined target mask size and limit, our R package can evaluate data collected using multiple mask sizes to detect the size most sensitive to morphological differences so that it can be used when processing test images. To take advantage of this, we computed masks using target sizes ranging from 200 to 800 µm^2^ inclusive at intervals of 100 µm^2^ (within a limit of ±100 µm^2^) for cells in our LPS dataset.

Smaller target sizes increase the number of cells that can be analysed as the masks are less likely to erroneously capture the morphology of adjacent cells and therefore face removal in the semi-automatic mask approval stage of our pipeline. During this stage, users are presented with each mask, overlaid on the original image of each cell, so that the mask can be visually inspected and rejected from further analysis if it extends onto other cells. Conversely, larger target sizes capture a greater extent of the cells' morphologies though at increased risk of extending onto other cells, and therefore increased risk of user rejection. By providing it with a range of target sizes, our R package can identify the mask size which balances a sufficient sample size with morphological sensitivity to optimally detect differences between training conditions.

To get as full a description of microglial morphology as possible, 62 morphological descriptors were derived from each cell mask, across five domains used previously to quantify microglial morphology [7,31]. These were shape descriptors (such as cell perimeter), skeleton analyses (for values such as the number of branches in a cell), Sholl analyses (for metrics based on the number of processes within a cell, such as the ramification index), hull and circularity morphometrics (HC morph; such as the convex hull area) and fractal analysis (for values such as lacunarity).

#### 3.3.3. Construction of a composite morphology metric

The data extracted by the ImageJ plugin was then used to build a single metric—the ‘Inflammation Index’—that was optimally sensitive to the morphological effects of LPS treatment with our R package (figure 3*a* and *b*).

#### 3.3.4. Mask size selection

First, we used the R package to identify the mask size associated with the greatest sensitivity to morphological changes in the LPS dataset. This required computing an inflammation index for each mask size, which we based on the work of Heindl *et al.* [9]. For each input mask size, the morphological descriptors best at discriminating between the training data (i.e. LPS) conditions are identified using a ROC-AUC analysis. While Heindl *et al.* [9] used a specific cutoff AUC value for selecting their best descriptors, we instead opted to use a cutoff based on their rank. As exact AUC values will differ depending on the quality of input images and the magnitude of the morphological changes associated with the training conditions, our approach allows greater generalizability across conditions, laboratories and experiments. If multiple variants of the same measurement are included in the chosen descriptors (e.g. some Sholl parameters are calculated for the 10th–90th percentiles of the data, as well as the whole dataset), the lowest performing are discarded. In addition, if highly correlated descriptors are included (e.g. we used a Pearson's correlation threshold of ≥ 0.9), the lowest performing are discarded. These cleaning steps ensure we do not overly weight our inflammation index to one morphological measure, or underlying factor.

To generate a single measure of morphological change, a PCA is then run on these best descriptors with the first principal component from the PCA being used as the inflammation index. A PCA combines the input metrics in ways that best capture the variability between cells by generating multiple components based on linear combinations of the inputs. It does this in an ordered manner i.e. the first principal component is the combination of metrics that describes the most variation between subjects, the second component explains the second largest portion of the variance, and so on. By selecting the first principal component, we are therefore selecting a single composite value that best enables us to distinguish between our subjects. The value of this component depends on what weightings have been applied to the input features, so its direction can vary between datasets (e.g. highly inflamed cells may have a positive value for one of our target mask sizes, but a negative value for another).

After generating an inflammation index for each mask size, the ability of each index to discriminate between training conditions for its mask size was evaluated using a ROC-AUC analysis. The mask size with the largest AUC value was identified as the optimal mask size. For our morphology data, this was 400 µm^2^ (figure 3*a*), though Kozlowski & Weimer [7] found 500 µm^2^ (with a limit of ±100 µm^2^) provided maximal cell detection with good sensitivity to LPS-induced morphological changes. This is likely to differ across set-ups and experiments.

#### 3.3.5. Inflammation index optimization

Having defined the optimal mask size, the inflammation index was then refined into its final form by identifying the combination of morphological descriptors (i.e. the best one through four, one through five, one through six, etc.) that led to the largest ROC-AUC value when using it to classify training conditions within the optimal mask size. For our data, this was the best two features (cell perimeter and the number of endpoint voxels in the skeletonized image of the cell mask). The pipeline also allows users to use the *p*-value as an alternative method for assessing the optimal combination of features. Our final inflammation index discriminated between our pre- and post-LPS training data with a standardized effect size of 1.08.

### 3.4. Applying the inflammation index to novel data

The identity and weightings of the discriminators in the final index were then used to generate an inflammation index for microglia in our test dataset, where we imaged CX3CR1-GFP^+/−^ mice fed a control diet or HFD after 56 days. These images were processed with the ImageJ plugin employing our optimal mask size (400 µm^2^ ± 100) before the R package was used to apply the inflammation index generated using the LPS data to calculate an inflammation index for each test cell, effectively quantifying how similar the morphology of our test cells were to LPS-activated microglia.

Our results showed no effect of HFD feeding on microglial morphology (figure 3*c*; control mean ± s.d. of 0.44 ± 1.66; HFD mean ± s.d. of 0.90 ± 1.47; *p* = 0.107). Though HFD has been shown to cause inflammation and activation of microglia in some strains of mice [27–29], the effects are expected to be weaker than those of LPS, which (unlike serum derived from mice fed a HFD for 16 weeks) significantly increases TNF alpha protein levels in isolated microglia [32]. Cx3CR1-GFP^+/−^ mice possess a heterozygous knockout of Cx3CR1 function, which has recently been shown to make them resistant to inflammation, including after HFD feeding [33], so use of these mice probably resulted in us not observing HFD-mediated microglial activation. Of note, abuse of the inflammation index, by using control and HFD microglia as a training dataset to create an index that is optimally sensitive to the test data, produces a highly significant difference in a statistical test of control (mean ± s.d. of −0.73 ± 1.74) versus HFD (0.55 ± 1.89) conditions (*p* = 0.0027; linear mixed model with the animal ID specified as a random intercept). This highlights the importance of using independent test and training treatment conditions.

#### 3.4.1. Further validating the inflammation index

**Figure 5.**
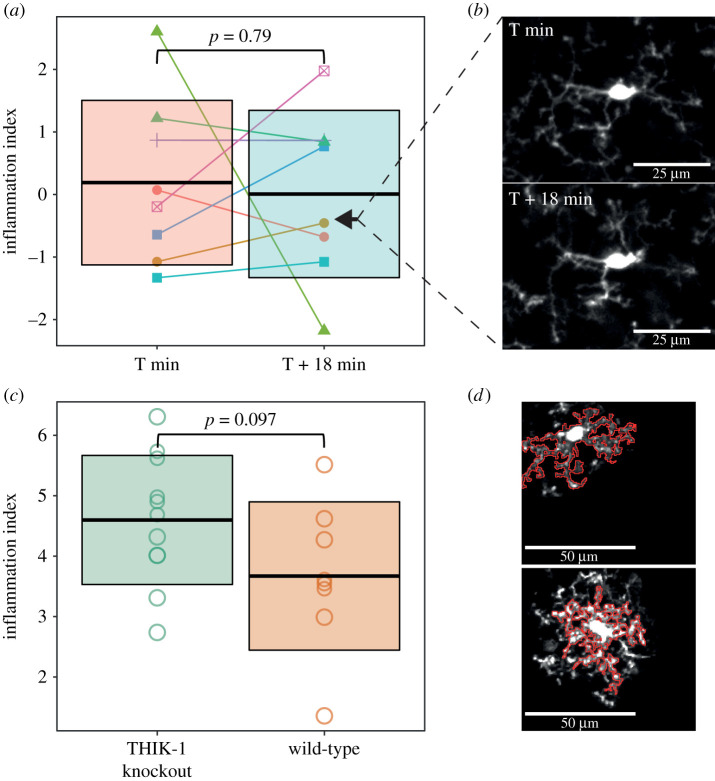
Further validation of the inflammation index. (*a*) Comparing the inflammation index between cells imaged 18 min apart revealed no significant effect of time on the value of the inflammation index (T+0 min, mean ± s.d. of 0.19 ± 1.32; T + 18 min, mean ± s.d. of 0.01 ± 1.34; *p* = 0.79; linear mixed model with animal ID specified as a random intercept; *n* = 8 cells, five animals. Crossbars represent the mean ± 1 s.d. Colour identifies individual cells, shape identifies individual animals. Arrowheads indicate the cell that is shown in (*b*). (*b*) Example images that were analysed, corresponding to the cell indicated by the arrowheads in (*a*). (*c*) Our analysis of THIK-1 knockout cells revealed a trend level effect on the inflammation index (wild-type mean ± s.d. of 3.66 ± 1.23; knockout mean ± s.d. of 4.58 ± 1.07; *p* = 0.097; one-way ANOVA, *n* = 8 cells for wild-type, *n* = 11 cells for knockout). (*d*) Example images of a wild-type and THIK-1 knockout cell with their automatically generated cell masks overlaid in red.

To further validate our pipeline and investigate the stability of the inflammation index for a given cell over a short timeframe, we also applied it to image stacks we captured for the purposes of analysing changes in microglial morphology within an imaging session. Relative to our primary morphology dataset, these images had a higher resolution in both *x* and *y*, but lower in *z*, and were acquired with greater scanning speed. As with our HFD analysis, we generated an inflammation index using images of control cells before and after their treatment with LPS, though here we subsequently applied it to images of cells collected the day before dietary manipulation began. These cells were imaged twice, 18 min apart. The inflammation index generated using this data included ten descriptors, far more than the two included in the index generated for the HFD analysis. The chosen descriptors included roundness, soma area, average branch length and circularity. This difference is probably down to the increased resolution in *x* and *y* giving our measurements greater sensitivity to morphological change and therefore greater ability to capture the effect of LPS treatment. There was no effect of time on this inflammation index (figure 5*a* and *b*; first stack mean ± s.d. of 0.19 ± 1.32; second stack mean ± s.d. of 0.01 ± 1.34; *p* = 0.79; linear mixed model with the animal ID specified as a random intercept), suggesting its value is stable for a given cell during an imaging session.

Additionally, we wanted to test the flexibility of our pipeline by applying it to data collected with a different imaging set-up, and for a different purpose. To do this, we applied it to *in vivo* microglial images from wild-type and THIK-1 knockout mice. These mice show reduced microglial process ramification, process number and process length [10]. The authors kindly provided a subset of the data used in this publication, but as this dataset lacked a positive control to use for training our pipeline, we instead trained it on the same pre- and post-LPS morphology dataset we used for our HFD analysis. This meant our index was less sensitive as both the optimal mask size and optimal metrics identified using our LPS dataset are likely to be sub-optimal choices for the THIK-1 dataset. However, our index was still, at trend level, higher in the knockout cells compared to wild-type cells (figure 5*c* and *d*; wild-type mean ± s.d. of 3.66 ± 1.23; knockout mean ± s.d. of 4.58 ± 1.07; *p* = 0.097; one-way ANOVA). This is consistent with the results reported by Madry *et al*. [10].

Given the inflammation index generated from our own images did not detect a significant difference in morphology in the THIK-1 data, we were interested in which parameters our pipeline would have identified as being optimally sensitive for doing so. We therefore used this THIK-1 knockout dataset to generate an index that was optimally sensitive to THIK-1 knockout-related morphological changes. Our pipeline identified nine metrics for inclusion (table 2) most of which reflect the reduced process number, length and complexity reported by Madry *et al*. [10]. For example, our index was built using the intersecting radii (the number of sampled radii with at least one intersecting process), the skewness of the distribution of the number of processes and the number of processes directly associated with the cell soma. All of these showed reduced mean values in our THIK-1 knockout cells and the nine metrics had an average AUC value of 0.76 when used in a ROC-AUC analysis to classify cells as either knockout or wild-type. Our index also identified soma area as an input metric, suggesting this may also be reduced by THIK-1 knockout. In comparison, the two metrics used to generate the index we analysed the THIK-1 dataset with (cell perimeter and the number of endpoint voxels in the skeletonized image of the cell mask) had an average AUC value of 0.59. While far below 0.76, this is better than chance and aligns with our ability to detect a morphological difference at the trend level. Interestingly, the optimal mask size detected for the THIK-1 dataset was 300 µm^2^, compared to a value of 400 µm^2^ for our LPS dataset. While this means the masks probably captured a lesser extent of the cells' morphologies, the fact that the THIK-1 images were captured at a higher resolution (*x* and *y* resolution of 0.39 µm compared to 0.58 µm) means that subtle morphological changes could be detected regardless.

**Table 2.** Morphological features included in the THIK-1 KO trained inflammation index. Each row indicates a morphological feature that was included in the inflammation index trained to detect differences in morphology between wild-type and THIK-1 knockout cells. Their mean values are displayed for the THIK-1 knockout and wild-type cells.

parameter	THIK-1 knockout	wild-type
triple points	22.25	25.73
CV for all radii	0.13	0.18
intersecting radii	56.50	66.82
max/min radii	1.59	1.76
maximum radius from hull's centre of mass (μm)	22.82	26.21
primary branches	2.19	3.73
regression intercept (log–log) [P10-P90]	2.27	3.80
skewness (sampled)	−0.04	0.34
soma area (μm^2^)	42.87	50.26

## 4. Conclusion

We present a fully open access analysis pipeline for analysing changes in microglial morphology in large *in vivo* imaging datasets. Our two packages, in ImageJ and R, provide a streamlined process for analysing complex morphological data and are available on GitHub to allow free access and encourage participation in their future development. The supplied packages can be applied to any experimental condition, e.g. detecting regional differences, simply by altering on the training data used. We anticipate our pipeline will greatly increase the ease through which multiple groups can take advantage of the power of *in vivo* microglial morphology analysis in a manner that is quick, streamlined, and preserves both statistical rigour and statistical power.

## Data accessibility

The data that support the findings of this study are openly available in Figshare at https://doi.org/10.6084/m9.figshare.14740719.v1, https://doi.org/10.6084/m9.figshare.14740725.v1, https://doi.org/10.6084/m9.figshare.14740731.v1, https://doi.org/10.6084/m9.figshare.14740740.v1, https://doi.org/10.6084/m9.figshare.14751084.v1.

## References

[1] Aloisi F. 2001 Immune function of microglia. Glia **36**, 165-179. (10.1002/glia.1106) 11596125

[2] Wolf SA, Boddeke HWGM, Kettenmann H. 2017 Microglia in physiology and disease. Ann. Rev. Physiol. **79**, 619-643. (10.1146/annurev-physiol-022516-034406) 27959620

[3] Tremblay MÈ, Stevens B, Sierra A, Wake H, Bessis A, Nimmerjahn A. 2011 The role of microglia in the healthy brain. J. Neurosci. **31**, 16 064-16 069. (10.1523/JNEUROSCI.4158-11.2011) PMC6633221 22072657

[4] Colonna M, Butovsky O. 2017 Microglia function in the central nervous system during health and neurodegeneration. Ann. Rev. Immunol. **35**, 441-468. (10.1146/annurev-immunol-051116-052358) 28226226 PMC8167938

[5] Nimmerjahn A, Kirchhoff F, Helmchen F. 2005 Resting microglial cells are highly dynamic surveillants of brain parenchyma in vivo. Science **308**, 1314-1318. (10.1126/science.1110647) 15831717

[6] Hierro-Bujalance C, Bacskai BJ, Garcia-Alloza M. 2018 In vivo imaging of microglia with multiphoton microscopy. Front. Aging Neurosci. **10**, 218. (10.3389/fnagi.2018.00218) 30072888 PMC6060250

[7] Kozlowski C, Weimer RM. 2012 An automated method to quantify microglia morphology and application to monitor activation state longitudinally in vivo. PLoS ONE **7**, e31814. (10.1371/journal.pone.0031814) 22457705 PMC3294422

[8] York EM, LeDue JM, Bernier LP, MacVicar BA. 2018 3DMorph automatic analysis of microglial morphology in three dimensions from *ex vivo* and *in vivo* imaging. eNeuro **5**, e0266-18.2018 1-12. (10.1523/ENEURO.0266-18.2018) PMC6325541 30627639

[9] Heindl S, Gesierich B, Benakis C, Llovera G, Duering M, Liesz A. 2018 Automated morphological analysis of microglia after stroke. Front. Cellular Neurosci. **12**, 106. (10.3389/fncel.2018.00106) PMC5917008 29725290

[10] Madry C, Kyrargyri V, Arancibia-Cárcamo IL, Jolivet R, Kohsaka S, Bryan RM, Attwell D. 2018 Microglial ramification, surveillance, and interleukin-1*β* release are regulated by the two-pore domain K^+^ channel THIK-1. Neuron **97**, 299-312. (10.1016/j.neuron.2017.12.002) 29290552 PMC5783715

[11] Jung S, Aliberti J, Graemmel P, Sunshine MJ, Kreutzberg GW, Sher A, Littman DR. 2000 Analysis of fractalkine receptor CX3CR1 function by targeted deletion and green fluorescent protein reporter gene insertion. Mol. Cellular Biol. **20**, 4106-4114. (10.1128/MCB.20.11.4106-4114.2000) 10805752 PMC85780

[12] Maggi L, Scianni M, Branchi I, D'Andrea I, Lauro C, Limatola C. 2011 CX3CR1 deficiency alters hippocampal-dependent plasticity phenomena blunting the effects of enriched environment. Front. Cellular Neurosci. **5**, 22. (10.3389/fncel.2011.00022) PMC3198035 22025910

[13] Shoffstall AJ, Paiz JE, Miller DM, Rial GM, Willis MT, Menendez DM, Hostler SR, Capadona JR. 2018 Potential for thermal damage to the blood–brain barrier during craniotomy: implications for intracortical recording microelectrodes. J. Neural Eng. **15**, 034001. (10.1088/1741-2552/aa9f32) 29205169 PMC6482047

[14] Hinwood M, Tynan RJ, Charnley JL, Beynon SB, Day TA, Walker FR. 2013 Chronic stress induced remodeling of the prefrontal cortex: structural re-organization of microglia and the inhibitory effect of minocycline. Cerebral Cortex **23**, 1784-1797. (10.1093/cercor/bhs151) 22710611

[15] Holtmaat A et al. 2009 Long-term, high-resolution imaging in the mouse neocortex through a chronic cranial window. Nat. Protocols **4**, 1128-1144. (10.1038/nprot.2009.89) 19617885 PMC3072839

[16] Schindelin J et al. 2012 Fiji: an open-source platform for biological-image analysis. Nat. Methods **9**, 676-682. (10.1038/nmeth.2019) 22743772 PMC3855844

[17] Čapek M, Janáček J, Kubínová L. 2006 Methods for compensation of the light attenuation with depth of images captured by a confocal microscope. Microsc. Res. Tech. **69**, 624-635. (10.1002/jemt.20330) 16741977

[18] Meijering E. 2003 Featurej: an ImageJ plugin suite for image feature extraction. See http://imagescience.org/meijering/software/featurej/.

[19] Thevenaz P, Ruttimann UE, Unser M. 1998 A pyramid approach to subpixel registration based on intensity. IEEE Trans. Image Process. **7**, 27-41. (10.1109/83.650848) 18267377

[20] Soulet D, Paré A, Coste J, Lacroix S. 2013 Automated filtering of intrinsic movement artifacts during two-photon intravital microscopy. PLoS ONE **8**, e53942. (10.1371/journal.pone.0053942) 23326545 PMC3543396

[21] Hanslovsky P, Bogovic JA, Saalfeld S. 2015 Post-acquisition image based compensation for thickness variation in microscopy section series. See https://arxiv.org/abs/1411.6970.

[22] Otsu N. 1979 A threshold selection method from gray-level histograms. IEEE Trans. Syst. Man Cybernetics **9**, 62-66. (10.1109/TSMC.1979.4310076)

[23] Longair M. 2006 Find Connected Regions ImageJ plugin. See https://imagej.net/Find_Connected_Regions.

[24] Ferreira TA, Blackman AV, Oyrer J, Jayabal S, Chung AJ, Watt AJ, Sjöström PJ, van Meyel DJ. 2014 Neuronal morphometry directly from bitmap images. Nat. Methods **11**, 982-984. (10.1038/nmeth.3125) 25264773 PMC5271921

[25] Arganda-Carreras I, Fernández-González R, Muñoz-Barrutia A, Ortiz-De-Solorzano C. 2010 3D reconstruction of histological sections: application to mammary gland tissue. Microsc. Res. Tech. **73**, 1019-1029. (10.1002/jemt.20829) 20232465

[26] Karperien AL. 2013 FracLac for ImageJ. See https://imagej.nih.gov/ij/plugins/fraclac/fraclac.html.

[27] Thaler JP et al. 2012 Obesity is associated with hypothalamic injury in rodents and humans. J. Clin. Invest. **122**, 153-162. (10.1172/JCI59660) 22201683 PMC3248304

[28] Calvo-Ochoa E, Hernández-Ortega K, Ferrera P, Morimoto S, Arias C. 2014 Short-term high-fat-and-fructose feeding produces insulin signaling alterations accompanied by neurite and synaptic reduction and astroglial activation in the rat hippocampus. J. Cerebral Blood Flow Metabolism **34**, 1001-1008. (10.1038/jcbfm.2014.48) PMC4050245 24667917

[29] Denver P, Gault VA, McClean PL. 2018 Sustained high-fat diet modulates inflammation, insulin signalling and cognition in mice and a modified xenin peptide ameliorates neuropathology in a chronic high-fat model. Diab. Obesity Metabolism **20**, 1166-1175. (10.1111/dom.13210) 29316242

[30] Bansal R, Raj G, Choudhury T. 2016 Blur image detection using Laplacian operator and Open-CV. In 2016 Int. Conf. System Modeling & Advancement in Research Trends (SMART), pp. 63-67. Piscataway, NJ: IEEE.

[31] Fernández-Arjona MDM, Grondona JM, Granados-Durán P, Fernández-Llebrez P, López-Ávalos MD. 2017 Microglia morphological categorization in a rat model of neuroinflammation by hierarchical cluster and principal components analysis. Front. Cellular Neurosci. **11**, 235. (10.3389/fncel.2017.00235) PMC5550745 28848398

[32] Baufeld C, Osterloh A, Prokop S, Miller KR, Heppner FL. 2016 High-fat diet-induced brain region-specific phenotypic spectrum of CNS resident microglia. Acta Neuropathol. **132**, 361-375. (10.1007/s00401-016-1595-4) 27393312 PMC4992033

[33] Cope EC, LaMarca EA, Monari PK, Olson LB, Martinez S, Zych AD, Katchur NJ, Gould E. 2018 Microglia play an active role in obesity-associated cognitive decline. J. Neurosci. **38**, 8889-8904. (10.1523/JNEUROSCI.0789-18.2018) 30201764 PMC6181311

